# Histological characterisation of gonadal sex differentiation in Malabar red snapper ( *Lutjanus malabaricus* ) for aquaculture advancement

**DOI:** 10.1111/jfb.70411

**Published:** 2026-03-17

**Authors:** Bing Liang, Dean R. Jerry, Joyce Koh, Purushothaman Kathiresan, Celestine Terence, Xueyan Shen, Grace Loo, Shubha Vij, Jose A. Domingos

**Affiliations:** ^1^ Tropical Futures Institute James Cook University Singapore Singapore Singapore; ^2^ Marine Aquaculture Centre Singapore Food Agency Singapore Singapore; ^3^ ARC Research Hub for Supercharging Tropical Aquaculture through Genetic Solutions James Cook University Townsville Queensland Australia; ^4^ School of Applied Science Republic Polytechnic Singapore Singapore; ^5^ Department of Preclinical Sciences and Pathology, Faculty of Veterinary Medicine Norwegian University of Life Sciences Ås Norway

**Keywords:** gonadal development, *Lutjanus malabaricus*, Malabar red snapper, sex control, sex differentiation

## Abstract

The Malabar red snapper (*Lutjanus malabaricus*) is a high‐value tropical marine species receiving growing attention for aquaculture development in Singapore and Southeast Asia. At present, seed production relies primarily on uncontrolled mass spawning in sea cages, a practice that lacks consistency, biosecurity and control of genetic contributions. A clear understanding of gonadal sex differentiation is essential for designing effective sex control strategies and establishing selective breeding programmes. However, the progression of gonadal development from initial differentiation through to sexual maturity in *L. malabaricus* remains poorly characterized. In this study, a comprehensive histological investigation of gonadal differentiation was conducted by tracking individuals from 1 to 445 days post‐hatch (dph). Gonads first appeared as undifferentiated structures at 14 dph, located between the swim bladder and intestine. The onset of ovarian differentiation was observed at 77 dph, marked by ovarian cavity formation, while testicular differentiation commenced at 128 dph, indicated by the development of lobular testis structures. Oogenesis was initiated by 169 dph, marked by the first observation of oogonia, and progressed to the chromatin nucleolus oocyte stage by 249 dph. Spermatogenesis was first evident at 249 dph, based on the appearance of lobular organization and spermatogonia, and further progressed by 353 dph, when spermatocytes, spermatids and spermatozoa were observed. These observations establish the first developmental timeline of gonadal sex differentiation in *L. malabaricus*, identifying key morphological features that distinguish early‐stage ovaries from testes. Importantly, our study also revealed a highly skewed sex ratio in a harvest‐size cohort from a commercial farm, with females comprising over 85% of the population. Given the species is considered gonochoristic, this finding raises concerns about potential influences from environmental factors, hatchery practices or genetic bottlenecks, and highlights the need for further investigation under controlled conditions. Together, these findings provide essential biological benchmarks for identifying the timing of sex differentiation and suggest that the optimal windows for sex manipulation occur around 60 dph to promote testicular development and approximately 110 dph to promote ovarian development. This work lays the foundation for future sex control techniques and contributes valuable knowledge toward the development of selective breeding programmes and sustainable seed production in this emerging aquaculture species.

## 1 INTRODUCTION

A clear understanding of gonadal differentiation in fish is essential for advancing aquaculture through improved reproductive management and production efficiency. A key strategy widely adopted in commercial aquaculture is the use of monosex populations, which maximizes somatic growth by avoiding the energetic costs of reproductive development. The preferred sex for culture varies by species. For instance, all‐male populations are used in Nile tilapia (*Oreochromis niloticus*), as males grow faster and more uniformly, whereas females mature early and can cause unwanted reproduction and overcrowding (Beardmore et al., 2001; Lind et al., 2015; Lu et al., 2022; Madalitso et al., 2020). In contrast, all‐female populations are preferred in salmonids, such as rainbow trout (*Oncorhynchus mykiss*) and Atlantic salmon (*Salmo salar*), because males often undergo early sexual maturation, reducing growth and flesh quality, while females maintain superior performance and delayed maturation (Piferrer, 2001).

While monosex culture is an important research focus for improving growth in fish reared to harvest size for human consumption where uncontrolled reproduction occurs or sexual dimorphism in commercially valuable traits exist, insights into gonadal differentiation are also crucial for broodstock fish maintained for larval production, where balanced sex ratios underpin reliable spawning and effective breeding programme management. In captive environments, sex ratios can become skewed due to environmental factors such as temperature or endocrine disruptors, undermining reproductive output and genetic diversity (Baroiller & D'Cotta, 2016). Early identification and manipulation of sex can be used to maintain a functional ratio of males and females for optimal spawning outcomes. Additionally, knowledge of gonadal development is essential for managing sexual maturation, particularly the timing and progression of puberty in cultured fish. For example, early or uncontrolled sexual maturation can divert energy from somatic growth and reduce flesh quality in grow‐out fish, whereas delayed maturation can limit larval production and slow the progress of selective breeding programmes.

Beyond aquaculture, knowledge of gonadal development also plays a vital role in conservation efforts and stock enhancement programmes for wild fisheries. Sex differentiation research can inform restocking programmes by revealing how environmental stressors or population structure influence sex ratios and reproductive capacity, as demonstrated in European sea bass (*Dicentrarchus labrax*) (Geffroy et al., 2021). Furthermore, fish serve as powerful models for understanding vertebrate sex differentiation due to their diversity of sex‐determining systems, ranging from genetic to environmental and polygenic mechanisms (Capel, 2017; Heule et al., 2014). This diversity offers a valuable comparative framework for studying the evolution of sex‐related genes and pathways.

The Malabar red snapper (*Lutjanus malabaricus*) is a gonochoristic tropical marine species of high economic importance in Southeast Asia and a key target for aquaculture expansion in Singapore. Its firm texture, market appeal and adaptability to captive rearing make it a promising candidate for intensive production systems. However, unlike more established aquaculture species such as Nile tilapia and Atlantic salmon, which benefit from advanced hatchery and effective sex control protocols (Wang, 2019), red snapper farming remains technologically underdeveloped. Its egg production still relies on mass spawning in open sea cages with minimal control over broodstock reproduction. This uncontrolled approach compromises biosecurity, limits pedigree traceability and leads to inconsistent seed quality. Compounding this issue is a skewed sex ratio in the farmed population in Singapore, with a predominance of females, which poses challenges for broodstock planning and selective breeding.

Recent efforts have focused on harvest traits and genomic resources for *L. malabaricus* (Liang et al., 2024, 2025; Purushothaman et al., 2024, 2025). Studies of wild‐caught individuals indicate that sexual maturity is typically reached at around 240 mm standard length (SL) in males and 250–300 mm SL in females, representing roughly 32%–50% of their maximum size, which is 645 mm SL in northern Australia and 740 mm SL in eastern Indonesia (Fry et al., 2009). Spawning peaks occur from September to March in northern Australia and in January–March and October in eastern Indonesia (Fry et al., 2009). Despite this, detailed knowledge of the species' reproductive biology, particularly the cellular and temporal progression of gonadal development, remains limited. Similar gaps exist for other red snapper species used in aquaculture, such as the northern red snapper (*Lutjanus campechanus*), for which most research efforts have focused on hatchery production (Buchalla et al., 2023), larval nutrition (Saillant et al., 2012) and grow‐out performance (McGuigan et al., 2023), rather than gonadal development. Understanding the histological progression of sex differentiation is essential to identify the labile period (e.g. the window during which sex can still be influenced by external factors such as hormonal treatment or temperature). In most teleosts, gonadal development begins with the specification and migration of primordial germ cells to the gonadal ridge during early larval stages. These cells then proliferate and contribute to the formation of a bipotential gonad, which later differentiates into an ovary or testis. The timing and morphological progression of gonadal differentiation differ markedly among species, influencing the applicability and design of sex manipulation strategies in aquaculture. Thus, identifying the sequence and timing of gonadal differentiation is fundamental to understanding how sex is established in this species. Histology remains the gold standard for documenting this process, allowing researchers to pinpoint the onset of sexual differentiation and the development of testicular or ovarian structures.

This study characterized the histological development of gonads in Malabar red snapper from early larval stages to sexual maturity. Through serial sampling across key developmental time points, we documented the morphological progression of gonadal structures, identified the onset and duration of the labile period, and generated essential data to guide the design of sex control strategies. These findings will support the development of sex control approaches for future breeding programmes and contribute to broader efforts to enhance the productivity and sustainability of red snapper aquaculture in Southeast Asia.

## 2 MATERIALS AND METHODS

### 2.1 Ethics statement

The experiment was conducted at James Cook University in Singapore under the approval of Institutional Animal Care and Use Committee (IACUC) number 2021‐A010.

### 2.2 Experimental design and sample collection

A preliminary study was conducted by collecting Malabar red snapper of various sizes from local farms and the Marine Aquaculture Centre (MAC) in Singapore. Histological assessment from this internal study indicated that the sex differentiation was completed in fish of 185 days post‐hatch (dph; unpublished data). Based on this, a systematic sampling scheme was implemented. The experimental cohort of *L. malabaricus* was produced via mass spawning involving multiple males and females at a commercial hatchery. Consequently, the sampled individuals were of mixed parentage and no pedigree or genetic information was available. The cohort was reared at MAC beginning on 6 September 2022, following standard larviculture protocols. After complete metamorphosis and weaning onto pelleted feeds, fish were stocked in a 3 m^3^ tank and progressively transferred to larger tanks up to 10 m^3^ as they increased in size, with stocking density maintained below 10 kg m^−3^. Rearing conditions were maintained at 29–31°C, pH 7.9–8.2, salinity 27–30 ppt, a 12 h light:12 h dark photoperiod, and dissolved oxygen levels above 5 ppm. Fish were fed commercial pellets containing 43–44% crude protein twice daily to apparent satiation. Systematic sampling was conducted over a period of more than 1 year from this single cohort, maintained in the same tank at any given time, to capture the full developmental trajectory of gonadal differentiation. Due to the small body size of fish during early ontogeny, different sampling strategies were employed at different stages. Whole‐body samples were collected at 1, 8 and 14 dph, while trunk sections containing the presumptive gonadal region were collected at 21, 28, 35 and 46 dph, as the fish were still too small to allow precise gonad dissection. From 64 dph onwards, dissected gonads were obtained for analysis. At each time point, six to 20 individuals were sampled. To ensure detailed monitoring of gonadal development, fortnightly sampling was performed between 64 and 144 dph. Thereafter, monthly sampling was carried out until 445 dph, extending well beyond the estimated completion of sex differentiation at approximately 185 dph based on our preliminary studies, to ensure full coverage of gonadal differentiation. This longitudinal sampling scheme enabled the capture of both early and late events in gonadal development, including the presumptive labile period during which sex differentiation is most responsive to environmental or hormonal cues. To preserve tissue integrity for histological analysis, whole fish, trunk sections or dissected gonads were fixed in 10% neutral buffered formalin containing 4% formaldehyde. Sampled fish were euthanized using an overdose of Aqui‐S® (100 ppm). All specimens were then fixed immediately following euthanasia for a minimum of 24 h prior to routine histological processing. In May 2023, a separate sampling was conducted at a commercial farm (Farm A) in Singapore to assess the sex ratio and body weight of harvest‐size Malabar red snapper aged approximately 1.5 years. Rearing conditions at Farm A were comparable to those at MAC, as described above. Gonadal tissues were collected from a total of 439 individuals. Sex was determined through gross morphological examination of the gonads in 267 fish, while the remaining 172 individuals with ambiguous gonadal appearance underwent histological analysis using the same fixation and processing protocols described above. Body weight was recorded for all sampled fish using a digital weighing balance, except for individuals at 14 and 77 dph. At 14 dph, only total length was measured due to their small size, while no morphometric data were available for 77 dph due to unintentional data loss during transfer. However, morphometric variation at this early juvenile stage is generally minimal across batches reared under similar culture conditions, making this data point less critical compared to later life stages where batch‐specific growth differences become more pronounced.

### 2.3 Gonadal histology

To ensure small larvae or gonadal tissues remained securely positioned during histological processing, samples were first embedded in agarose within tissue cassettes. To optimize resource utilization and reduce processing costs, multiple specimens (<347 dph) collected at the same sampling time point were size‐sorted and two to five individuals with similar body weights were embedded together in a single tissue cassette. The range of body weights for each group was reported to represent the sample weight distribution. Specimens ≥347 dph were processed individually to enable accurate sex determination and assessment of sex ratio. Whole‐body and trunk samples were subjected to decalcification to soften skeletal structures and facilitate sectioning. Histological preparation began with dehydration of the fixed tissues using increasing concentrations of ethanol, followed by clearing in xylene and subsequent embedding in paraffin wax. Serial sections of 5 μm thickness were cut using a rotary microtome and mounted onto glass microscope slides. The mounted sections were then dewaxed in xylene, rehydrated through a descending ethanol series, and stained with haematoxylin and eosin to visualize cellular morphology and structural details of the gonadal tissue (Purushothaman et al., 2016). Following staining, slides were dehydrated again through an ascending ethanol series, cleared in xylene and permanently mounted with coverslips. Prepared slides were examined using an Olympus BX53 System Microscope to document morphological changes in the developing gonads across all sampled time points.

### 2.4 Statistical analyses and data visualization

All statistical analyses were conducted using R version 4.5.1 within RStudio version 2025.05.1 (R Core Team, 2025). Differences in body weight between male and female Malabar red snapper from Farm A were assessed using Welch's two‐sample *t*‐test, which does not assume equal variance between groups. To determine whether the observed sex ratio at Farm A significantly deviated from an expected 1:1 distribution, a chi‐square (*χ* ^2^) goodness‐of‐fit test was applied. Statistical significance was set at *p* < 0.05 and results were reported using standard notation: *p < 0.05, **p < 0.01 and ***p < 0.001. Data visualization was performed using the *ggplot2* (Wickham, 2016) and *ggpubr* (Kassambara, 2020) packages. Sex information for MAC specimens ≥347 dph (*n* = 105) was obtained from histology slides to complement the sex ratio analysis conducted for Farm A.

## 3 RESULTS

**FIGURE 1:**
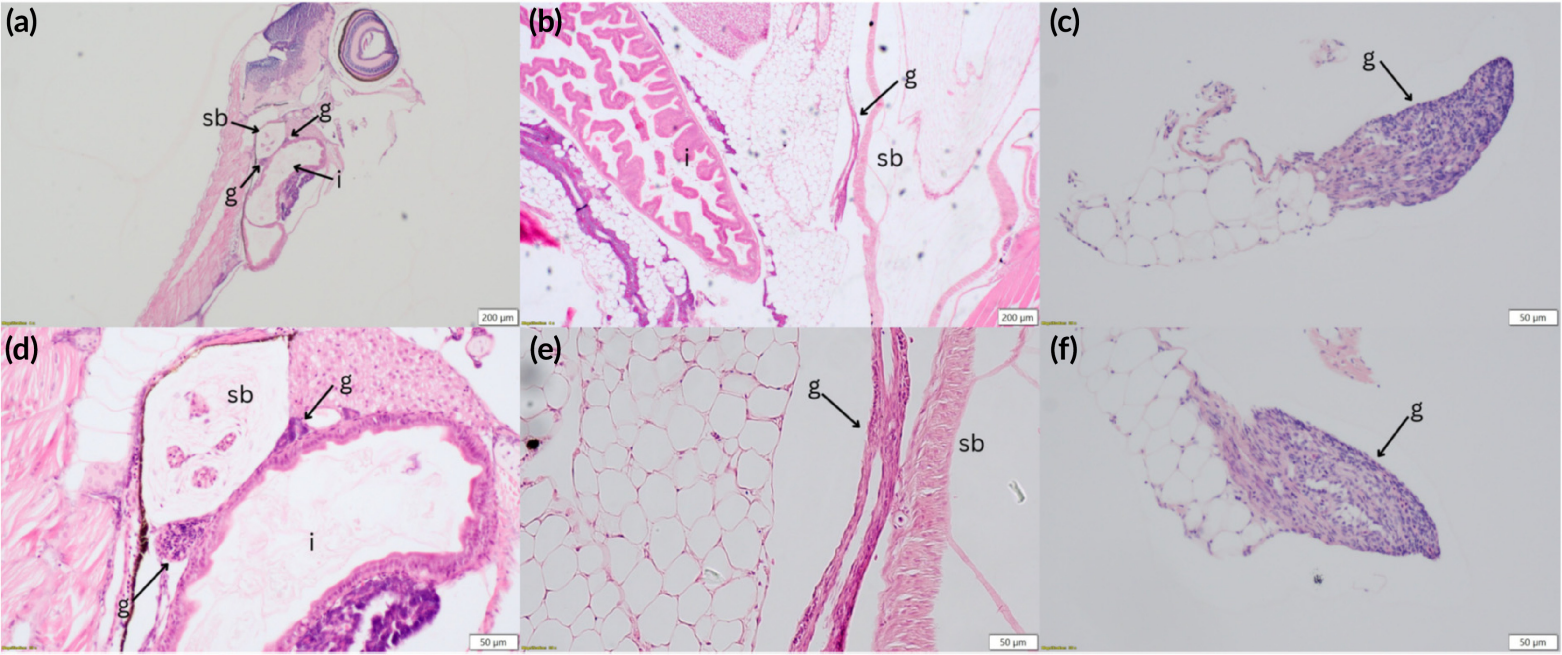
Early development of undifferentiated gonads in Malabar red snapper (*Lutjanus malabaricus*). (a–c) The location and morphology of the gonads at 14, 46 and 64 days post‐hatch (dph), respectively. (f) An additional example of a gonad at 64 dph. (d, e) Higher magnification views of the gonadal regions shown in (a) and (b), respectively, highlighting cellular detail. All histological sections were stained with haematoxylin and eosin. g, gonad; i, intestine; sb, swim bladder.

Representative histological images illustrating the progressive development of undifferentiated gonads in Malabar red snapper across various early life stages are presented in Figure 1. The presumptive gonadal tissue was first detected at 14 dph, located in the typical anatomical position between the swim bladder and the gut, as visualized in longitudinal sections of whole‐larval samples (Figure 1a,d). At this stage, the gonad appeared as a small, elongated structure with homogenous cellular composition and no visible signs of sex differentiation. As development progressed, the gonads increased in size and cellular complexity. By 46 dph, the gonadal region had expanded and displayed a more clearly defined structure, although still undifferentiated (Figure 1b,e). Further enlargement and tissue organization were observed at 64 dph, as shown in both transverse and longitudinal sections (Figure 1c,f), indicating ongoing growth and morphogenesis during the pre‐differentiation phase.

**FIGURE 2:**
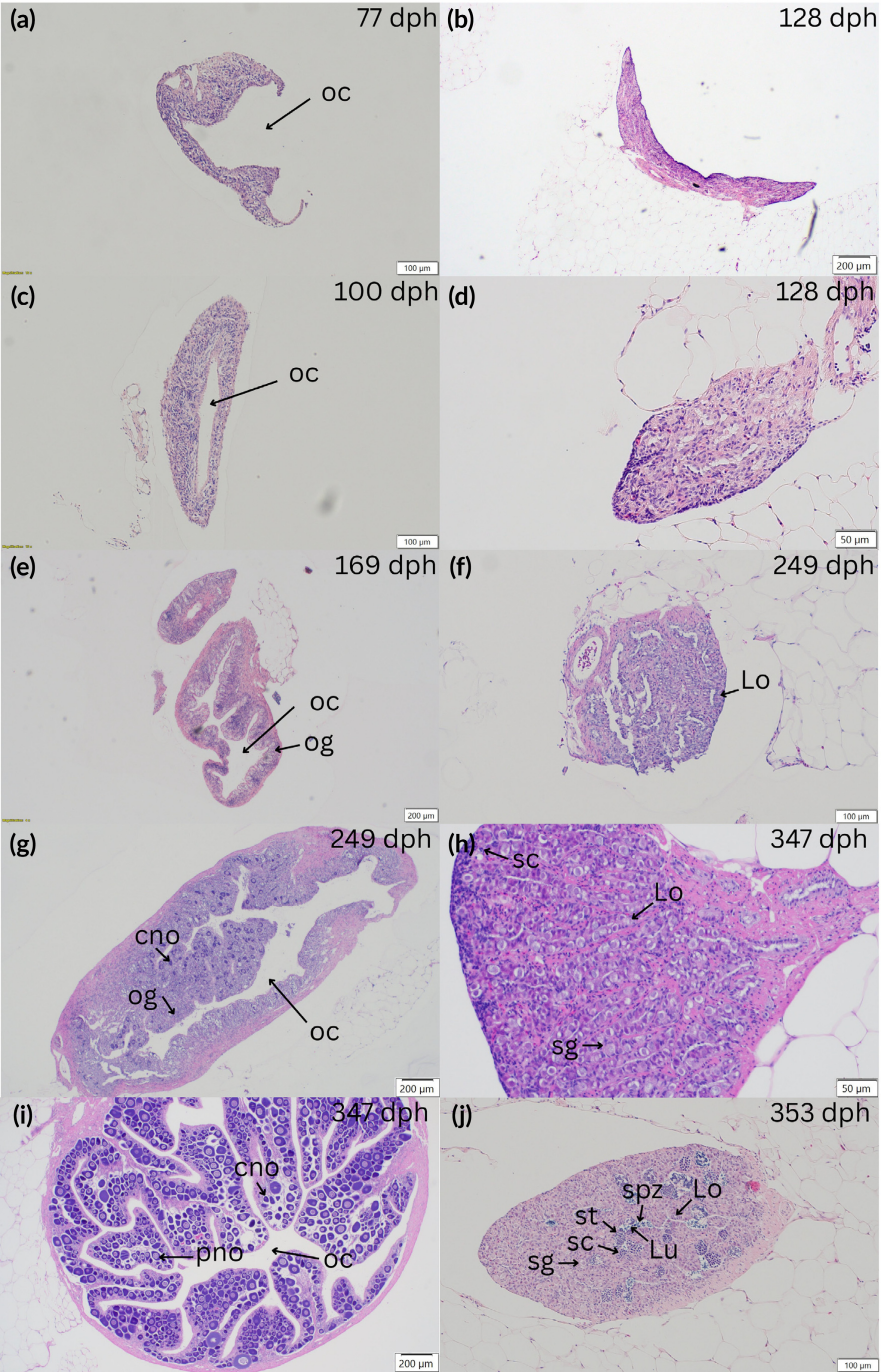
Development of differentiated gonads in Malabar red snapper (*Lutjanus malabaricus*). (a, c, e, g, i) The progressive development of female gonads at 77, 100, 169, 249 and 347 days post‐hatch (dph), respectively. (b, d, f, h, j) The morphological features of male gonads at 128 dph (longitudinal section in b and cross section in d), and at 249, 347 and 353 dph, respectively. All sections were stained with haematoxylin and eosin to highlight cellular structures involved in gonadal differentiation. cno, chromatin nucleolus oocytes; Lo, lobule; Lu, lumen; Oc, ovarian cavity; og, oogonia; pno, perinucleolar oocytes; sc, spermatocytes; sg, spermatogonia; spz, spermatozoa; st, spermatids.

**FIGURE 3:**
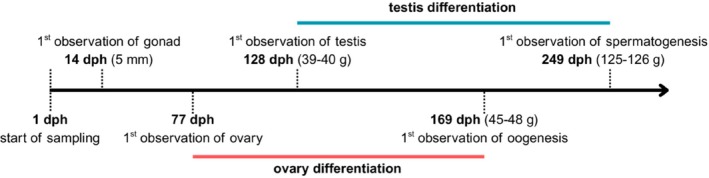
Developmental timeline of sex differentiation in Malabar red snapper (*Lutjanus malabaricus*), spanning from early gonadal dimorphism to the onset of gametogenesis. Time points are shown alongside corresponding total length for early‐stage fish (14 days post‐hatch [dph]; length data only) and body weight for later stages. Morphometric data for 77 dph were not available. Key histological milestones in ovarian and testicular differentiation are indicated along the timeline.

Histological images illustrating the process of gonadal differentiation in *L*. *malabaricus* are presented in Figure 2. The earliest morphological sign of ovarian differentiation was observed at 77 dph, marked by the formation and invagination of the ovary cavity, a key event signalling the commitment of the undifferentiated gonad toward female development (Nakamura et al., 1998). By 100 dph, the ovary cavity had fully enclosed and the gonad showed noticeable growth in size, further supporting its differentiation into an ovary. Continued ovarian development was evident as the fish grew. By 169 dph, the ovaries had further increased in size and oogonia were observed, marking initiation of oogenesis. Another developmental milestone was reached at 249 dph, when chromatin nucleolus oocytes were identified for the first time. These oocytes, representing the primary growth stage of oogenesis, are characterized by a large nucleus with one or more prominent nucleoli, sparse cytoplasmic content and the absence of yolk deposition, indicating the onset of early oocyte growth well before vitellogenesis and reproductive maturity. In contrast, male gonadal differentiation was first detected later, at 128 dph. The distinguishing feature of testicular differentiation at this stage was the formation of lobular structures, an important morphological hallmark of developing testes. As the fish aged, these lobules became increasingly distinct and testicular tissue expanded in volume. By 249 dph, the testis showed clear structural organization, with identifiable lobular tissue containing primary spermatogonia located at the periphery of the gonad, marking the initiation of spermatogenesis. By 353 dph, multiple differentiated germ cell types, including spermatogonia, spermatocytes, spermatids and spermatozoa, were clearly visible within the seminiferous lobules, indicating a functionally maturing testis. For all samples collected up to 445 dph, the most advanced gonads were classified as Maturity Stage II (Maturing), and no individuals reached Maturity Stage III (Mature) according to the criteria established in our previous study (Liang et al., 2024). Specifically, females had not initiated vitellogenesis and egg maturation and male gonads had not progressed to a stage dominated by spermatocytes and spermatids with the presence of spermatozoa. The complete timeline of sex differentiation in Malabar red snapper, including the corresponding total length for samples at 14 dph and body weight for all other time points, is summarized in Figure 3. This timeline spans from the earliest histological signs of gonadal dimorphism to the onset of gametogenesis.

**FIGURE 4:**
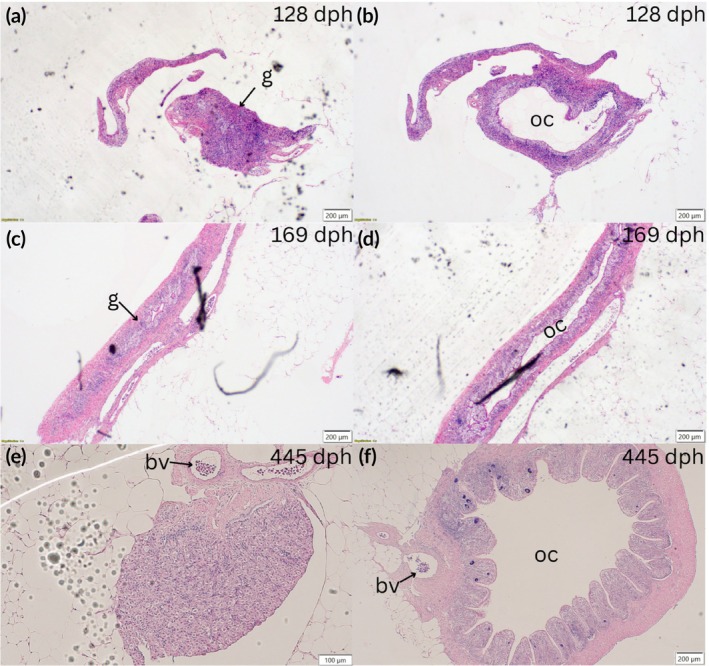
Impact of sectioning depth on the histological appearance of early gonads in Malabar red snapper (*Lutjanus malabaricus*). (a, c, e) Cross‐ or longitudinal sections of the same gonads as in (b, d, f), respectively, but at different tissue depths. All sections were stained with haematoxylin and eosin. bv, blood vessel; G, gonad; oc, ovary cavity.

Sections cut at different depths of the same embedded gonad block exhibited marked variation in histological appearance. Representative misleading sections from ovaries lacking an obvious ovarian cavity are shown in Figure 4. For example, Figure 4a,c,e were taken from the same gonads as Figure 4b,d,f, respectively, but at different depths within the paraffin block. In these shallower or deeper sections, the ovarian cavity is either absent or unobvious, creating a misleading resemblance to testicular tissue. These observations highlight the importance of examining multiple sections at different cutting depths to obtain an accurate interpretation of gonadal development. In addition, careful assessment of diagnostic features, particularly the presence of oogonia in ovaries or lobular organization in testes, aids in confirming gonadal identify.

**FIGURE 5:**
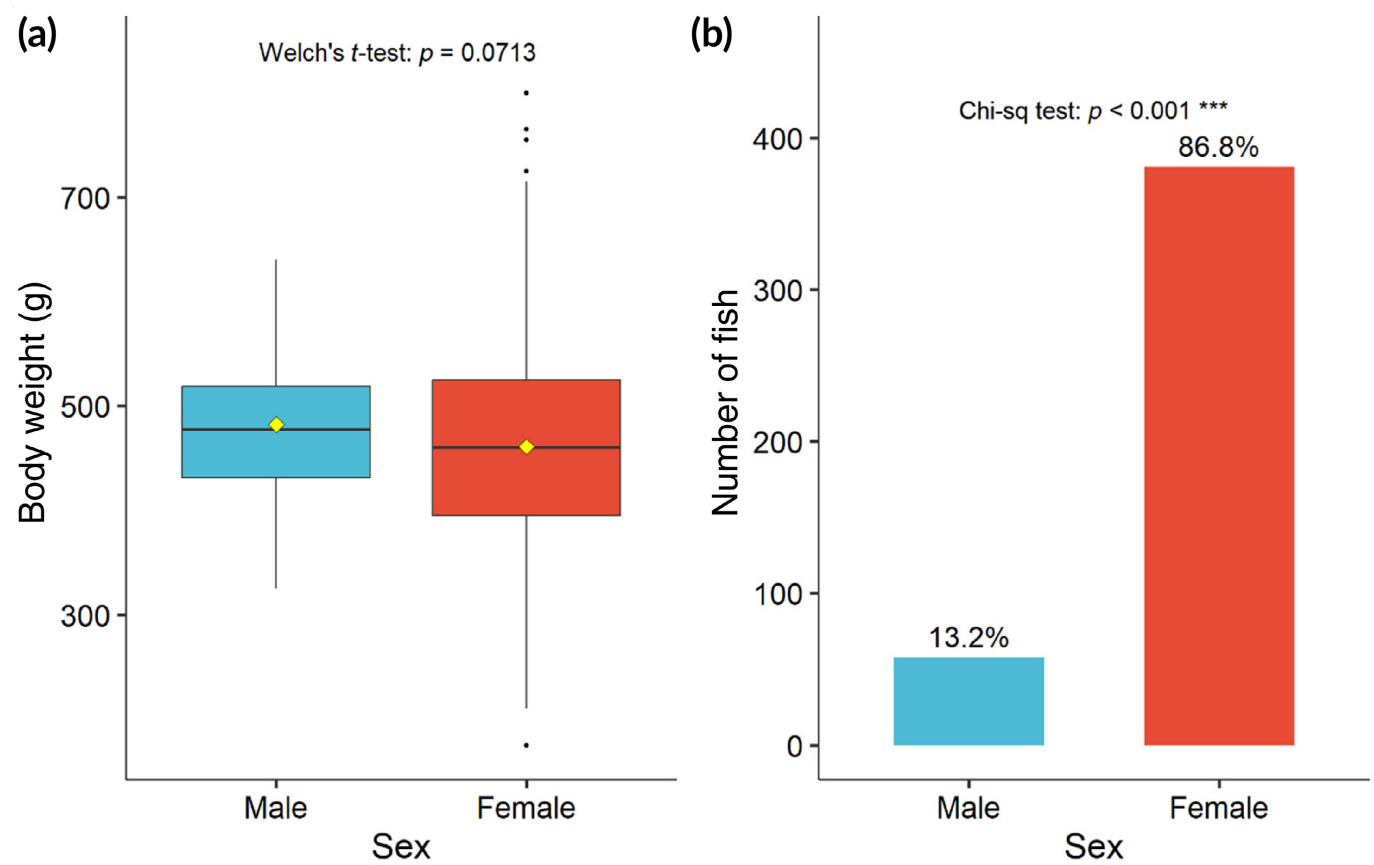
Sex‐specific body weight distribution and sex ratio of 1.5‐year‐old Malabar red snapper (*Lutjanus malabaricus*) at harvest size at Farm A. (a) Boxplot showing the distribution of body weights for male and female fish. The box represents the interquartile range, the horizontal line indicates the median, whiskers extend to 1.5× the interquartile range and yellow diamonds represent group means. A Welch's *t*‐test was performed to compare mean body weights between sexes; the *p* value and significance level are indicated. (b) Bar plot showing the number and proportion of males and females in the sampled population. Percentages are shown above each bar. A chi‐square (*χ* ^2^) goodness‐of‐fit test was used to assess deviation from a 1:1 sex ratio, with the significance level annotated. Significance is denoted as ***p < 0.001.

At harvest size, no statistically significant difference in body weight was observed between male and female Malabar red snapper (Figure 5a). While females showed slightly lower median and mean body weights than males, Welch's *t*‐test indicated that the difference was not significant (*p* = 0.0713). In contrast, sex ratio analysis revealed a highly significant deviation from the expected 1:1 distribution (Figure 5b). Out of 439 fish sampled, 86.8% were identified as female and only 13.2% as male. A *χ* ^2^ goodness‐of‐fit test confirmed this deviation was highly significant (***p < 0.001), indicating a strong female bias in the population at harvest. Sex data for MAC specimens ≥347 dph are summarized in Table 1, which likewise showed a marked female bias (76.2%) and aligned with the skewed sex ratio observed at Farm A. Throughout this study, no intersex or transitional gonadal stages (i.e. gonads exhibiting both ovarian and testicular tissue) were observed in any samples collected from MAC and Farm A. This supports the conclusion that *L. malabaricus* is a gonochoristic species in which individuals differentiate directly into either males or females. In addition, substantial variation in body size was observed among fish of the same age. For example, fingerlings at 128 dph exhibited body weights ranging from 19 to 53 g, with a mean of 31 g and a standard deviation of 10 g, highlighting pronounced inter‐individual growth variability within the cohort.

**TABLE 1:** Distribution of Malabar red snapper (≥347 days post hatch, dph) sampled at marine aquaculture centre (MAC) by sex and sampling age.

Sex	347 dph	353 dph	382 dph	421 dph	445 dph	Total (*n* (%))
Female	17	4	9	12	38	80 (76.2)
Male	6	4	4	2	7	23 (21.9)
Unknown	2	–	–	–	–	2 (1.9)
Total	25	8	13	14	45	105 (100)

*Note*: Sex was determined from histological examination of gonadal tissue.

## 4 DISCUSSION

The present study provides the first detailed histological timeline of gonadal development and sex differentiation in *L. malabaricus*, offering key insights into the morphological changes that underpin sexual differentiation in this commercially important marine species. The comprehensive sampling strategy, spanning from larval stages to early gametogenesis, allowed us to characterize the progression of undifferentiated gonads into functionally distinct ovaries and testes. A significant outcome of this study is the identification of the onset and sequence of gonadal differentiation, with ovarian and testicular pathways diverging as early as 77 and 128 dph, respectively. Although no published information is currently available on the timing of gonadal differentiation for Lutjanids, the present findings are consistent with reports in other fish species, where the timing of sex differentiation varies but follows a similar pattern of earlier ovarian development. For example, differentiation of ovaries and testes occurs at approximately 55 and 65 dph in the chameleon goby (*Tridentiger trigonocephalus*) (Cho et al., 2014), 49 and 69 dph in green sunfish (*Lepomis cyanellus*) (Teal et al., 2022), 101 and 150 dph in greater amberjack (*Seriola dumerili*) (Papadaki et al., 2021), 70 and 80 dph in bluegill sunfish (*Lepomis macrochirus*) (Gao et al., 2009), and 60 and 90 dph in turbot (*Scophthalmus maximus*) (Zhao et al., 2017). In protandrous species such as barramundi (*Lates calcarifer*), testicular differentiation begins even earlier, at around 44 dph (Banh et al., 2017).

In many fish species, the most effective period for inducing artificial sex change using exogenous steroid hormones coincides with the window of gonadal sex differentiation (Wang, 2019). Based on our histological observations, the optimal timing for sex manipulation in *L*. *malabaricus* appears to be prior to the onset of morphological differentiation: specifically, administering androgens around 60 dph could potentially promote development toward testicular fate before ovarian differentiation begins at 77 dph, while administering oestrogens around 110 dph may promote ovarian differentiation in individuals that remain undifferentiated at this stage before testicular differentiation initiates at 128 dph. For individuals in which ovarian differentiation had already occurred by 110 dph, oestrogen treatment may reinforce or stabilize ovarian development (Li et al., 2019).

The initiation of oogenesis in Malabar red snapper was observed significantly later (169 dph) than the onset of ovarian differentiation (77 dph). This temporal separation between the differentiation of somatic gonadal structures and the appearance of germ cells is consistent with previous findings in other tropical marine species such as the orange‐spotted spinefoot (*Siganus guttatus*) (Komatsu et al., 2006). By contrast, in some temperate freshwater species like the amago salmon (*Oncorhynchus rhodurus*), oogenesis occurs concurrently with the morphological differentiation of the ovary (Nakamura & Nagahama, 1993). These interspecies differences suggest that the timing of germ cell maturation relative to somatic gonadal differentiation may be regulated by species‐specific developmental and hormonal mechanisms rather than solely by environmental or habitat factors. This finding highlights the importance of species‐specific histological timelines for informing strategies in reproductive management and sex control in aquaculture.

One of the key morphological distinctions between developing ovaries and testes lies in the presence and organization of the ovary cavity. In females, the defining structure is a well‐formed ovary cavity, a large central lumen generated by the invagination and enclosure of the gonad epithelium. In contrast, developing testes lack a true central cavity and typically exhibit a compact tissue structure; any small, slit‐like empty spaces observed are most likely histological artefacts rather than anatomical lumens. In ovaries, small luminal spaces may occasionally be observed outside the main gonad mass, which can be misleading. However, careful examination reveals that these spaces are located within surrounding somatic tissue rather than within the gonad itself, distinguishing them from testicular structures. These structural differences serve as reliable markers for sex identification, especially during intermediate stages, when germ cell types may not yet be morphologically distinct.

The present findings highlight that the histological appearance of gonads can vary considerably depending on the plane and depth of tissue sectioning. As shown in Figure 4, sections taken too shallow or too deep within the same gonad block may obscure diagnostic features such as the ovarian cavity, creating ambiguous profiles that resemble testicular morphology. To minimize misclassification, multiple sections should be prepared at evenly distributed depths throughout each embedded gonad block, thereby providing a more representative view of gonadal architecture (Longenecker et al., 2020). This is particularly critical during early stages of sex differentiation, when ovarian cavities or testicular lobules may be underdeveloped or visible only in restricted regions of the gonad. Serial sectioning combined with careful contextual interpretation is therefore essential for accurate histological sex determination and for drawing reliable conclusions on sex ratios and developmental patterns in *L. malabaricus* and other fish species.

The testicular structure observed in *L. malabaricus* conforms to the lobular testis type, which is characteristic of higher teleost taxa (Parenti & Grier, 2004). In contrast to the tubular testis type often seen in more basal fish groups, the lobular architecture features discrete lobules radiating from the efferent duct system. Moreover, the testis of *L. malabaricus* is classified as an “unrestricted lobular testis” based on the spatial distribution of germ cell stages (Grier, 1981). Rather than displaying a strict maturation gradient, germ cells at various developmental stages—including spermatogonia, spermatocytes, spermatids and spermatozoa—are distributed throughout the testicular tissue. In addition, ovarian development in *L. malabaricus* is classified as asynchronous (Wallace & Selman, 2015), with oocytes at all developmental stages present without a clearly dominant cohort, as illustrated in Figure 1 of our previous study (Liang et al., 2024). This asynchronous pattern is typical of warmwater species capable of multiple or continuous spawning events throughout the year (Lowerre‐Barbieri et al., 2023; Lubzens et al., 2010). Indeed, *L. malabaricus* has been documented as an asynchronous spawner in the wild (Fry et al., 2009), and our histological findings are consistent with this reproductive strategy.

Given the substantial variation in body size among fish of the same age, and because both age and size are key factors influencing gonadal development (Chen et al., 2022), considerable inter‐individual variability in the timing of differentiation was anticipated. This variation in gonadal development may have been amplified by possible genetic and/or physiological differences, even among fish of similar age and size. Consequently, the oldest fish with undifferentiated gonads was recorded at 169 dph, weighing between 85 and 91 g—substantially larger than the earliest case of gonadal differentiation, observed at 77 dph in a fish from the same cohort. This disparity suggests that gonadal development is not strictly determined by chronological age but is influenced by differences in growth trajectories, genetic background or other intrinsic factors. These findings underscore the importance of considering individual variation, as well as both age and somatic growth, when assessing sexual development and developing effective sex control strategies.

This study confirmed that Malabar red snapper is gonochoristic, as no histological evidence of transitional gonadal stages—such as the simultaneous presence of male and female tissue—was observed in any of the hundreds of samples examined over more than a year of regular sampling. This finding is consistent with previous studies on other lutjanid species, where gonochorism has been consistently reported (Heupel et al., 2010; Newman et al., 2016). Furthermore, extensive histological surveys of wild populations of *L. malabaricus* have not documented cases of hermaphroditism or intermediate gonadal forms indicative of sex change (Fry et al., 2009; McPherson et al., 1992; Newman, 2002). Studies of wild populations from coastal waters of Vietnam indicate that first sexual maturity occurs at approximately 280–330 mm standard length in males and 230–280 mm in females. Spawning was observed throughout the year, with individuals in ripe or spawning condition recorded in most months, and distinct spawning peaks occurring in late March–April and August (Hai Yen et al., 2022). In the present study, the highly skewed sex ratios observed at both Farm A and in MAC specimens—substantially deviating from the expected 1:1 ratio—raise important questions. One possible explanation is exposure to endocrine disrupting chemicals in the water or feed, which may have induced feminisation. Similar effects have been reported as a potential cause of early sex change from male to female in barramundi farmed in Singapore (Terence et al., 2021). Another possibility is the pre‐harvest grading practices: since the fish were introduced to the farm as four‐inch fingerlings, they may have been selectively sorted by size, and sex‐based growth dimorphism could have resulted in a biased sex distribution. Additionally, genetic factors can also affect sex ratios as sex determination can be considered as a complex trait in fish because consistent family‐level variation in sex ratios has been reported in several fish species and additive genetic component underlying sex determination has been estimated (MartÃ­nez et al., 2014). Consequently, founder effects or sex‐linked inheritance patterns may have contributed to the observed sex ratio distortion, particularly if the farm stock originated from a limited number of broodstock carrying skewed sex genotypes or sex‐linked alleles. This risk is further amplified by the fact that red snapper seed production is predominantly concentrated in a few hatcheries in Malaysia, where broodstock genetic composition is typically not monitored or controlled. Lastly, environmental conditions or stressors experienced by broodstock (e.g. nutrition, density, temperature) may influence epigenetic regulation of sex differentiation in offspring, even in genetically fixed gonochorists. Interestingly, a previous study on *L. malabaricus* populations in Vietnam found that, although the overall sex ratio was 1:1, significant monthly fluctuations occurred (Hai Yen et al., 2022). For example, nearly 70% of individuals were female and 30% were male in December, potentially reflecting the combined influence of the factors discussed above. Therefore, to distinguish among these possibilities, future studies should assess sex ratios in ungraded populations of known origin, ideally comprising individuals older than 169 days post‐hatch and exceeding 91 g in body weight, when gonadal differentiation is complete. Controlled rearing experiments under uniform conditions are also needed to rule out environmental, nutritional or genetic influences on sex differentiation and confirm whether the observed skew is intrinsic or externally induced.

Although our initial aim included identifying the earliest stages of gonad formation, we were unable to observe primordial germ cells (PGCs) or the initial gonadal anlage prior to 14 dph. Despite extensive efforts—including pooling early‐stage larvae and performing both cross‐sectional and longitudinal histology at various tissue depths—PGCs were not detected in samples collected earlier than 14 dph. This limitation, however, does not compromise the main objective of the study, which was to define the labile period of sex differentiation. Future studies focusing specifically on the specification and migration of PGCs may consider collecting and analysing larval stages younger than 14 dph using specialized histological or molecular techniques such as immunostaining or in situ hybridisation targeting PGC markers.

## 5 CONCLUSIONS

This study provides the first comprehensive histological timeline of gonadal sex differentiation in Malabar red snapper, an emerging tropical aquaculture species. Gonads were first detected at 14 dph, with ovarian differentiation observed by 77 dph and testicular lobule formation by 128 dph. Oogenesis and spermatogenesis commenced at 169 and 249 dph, respectively. These findings suggest that the optimal windows for sex manipulation may lie around 60 dph to promote testicular development and around 110 dph to promote ovarian development. In addition to characterizing developmental milestones, this study also documented a highly skewed sex ratio in a farmed population, raising concerns about potential environmental, genetic or hatchery‐related influences on sex differentiation. This highlights the need for further investigation under controlled conditions to determine the underlying causes and ensure balanced sex ratios in breeding programmes. Together, these results establish critical developmental benchmarks for guiding sex control strategies and provide a strong foundation for future research aimed at improving seed production and advancing sustainable aquaculture of *L. malabaricus*.

## AUTHOR CONTRIBUTIONS

B.L.: Conceptualization, methodology, investigation, data analysis, writing–original draft preparation, review and editing. D.R.J. and J.A.D.: Conceptualization, methodology, data analysis, writing–review and editing, funding acquisition. X.S., G. L. and S.V.: Writing–review and editing, funding acquisition. P.K., J.K. and C.T.: Investigation, writing–review and editing. All authors gave final approval for publication.

## FUNDING INFORMATION

This work was funded by the Singapore Food Story (SFS) R&D Programme in ‘Sustainable Urban Food Production’ (NRF‐000190‐00; Proposal ID: SFSRNDSUFP1‐0097).

## CONFLICT OF INTEREST STATEMENT

The authors declare no competing interests.

## Data Availability

The data that support the findings of this study are available from the corresponding author upon reasonable request.
